# Point-of-Care Gastric Ultrasound Confirms the Inaccuracy of Gastric Residual Volume Measurement by Aspiration in Critically Ill Children: GastriPed Study

**DOI:** 10.3389/fped.2022.903944

**Published:** 2022-06-15

**Authors:** Frederic V. Valla, Eloise Cercueil, Claire Morice, Lyvonne N. Tume, Lionel Bouvet

**Affiliations:** ^1^Department of Pediatrics, Pediatric Intensive Care, Lyon University Children Hospital, Hospices Civils de Lyon, Lyon, France; ^2^Department of Pediatric Intensive Care Alder Hey Children's Hospital, School of Health & Society, University of Salford, Manchester, United Kingdom; ^3^Department of Anesthesiology and Intensive Care, Lyon University Children Hospital, Hospices Civils de Lyon, Lyon, France

**Keywords:** pediatric intensive care, critical care, feeding tolerance, enteral nutrition, gastric tube

## Abstract

**Introduction:**

No consensus exists on how to define enteral nutrition tolerance in critically ill children, and the relevance of gastric residual volume (GRV) is currently debated. The use of point-of-care ultrasound (POCUS) is increasing among pediatric intensivists, and gastric POCUS may offer a new bedside tool to assess feeding tolerance and pre-procedural status of the stomach content.

**Materials and Methods:**

A prospective observational study was conducted in a tertiary pediatric intensive care unit. Children on mechanical ventilation and enteral nutrition were included. Gastric POCUS was performed to assess gastric contents (empty, full of liquids or solids), and gastric volume was calculated as per the Spencer formula. Then, GRV was aspirated and measured. The second set of gastric POCUS measurements was performed, similarly to the first one performed prior to GRV measurement. The ability of GRV measurement to empty the stomach was compared to POCUS findings. Both GRV and POCUS gastric volumes were compared with any clinical signs of enteral feeding intolerance (vomiting).

**Results:**

Data from 64 children were analyzed. Gastric volumes were decreased between the POCUS measurements performed pre- and post-GRV aspiration [full stomach, *n* = 59 (92.2%) decreased to *n* = 46 (71.9%), *p* =0.001; gastric volume: 3.18 (2.40–4.60) ml/kg decreased to 2.65 (1.57–3.57), *p* < 0.001]. However, the stomach was not empty after GRV aspiration in 46/64 (71.9%) of the children. There was no association between signs of enteral feeding intolerance and the GRV obtained, nor with gastric volume measured with POCUS.

**Discussion:**

Gastric residual volume aspiration failed to empty the stomach and appeared unreliable as a measure of gastric emptiness. Gastric POCUS needs further evaluation to confirm its role.

## Introduction

Early enteral nutrition (EN) is recommended in critically ill children receiving invasive and non-invasive ventilation mechanical support (1). However, EN tolerance may be challenging in this setting by multifactorial gastroparesis and paralytic ileus (opioid use, bed rest, altered gut hormone secretion, plasmaelectrolyte disturbances, systemic inflammatory response syndrome) (2–4). The resulting increase in gastric volume may potentially lead to vomiting and aspiration and result in ventilation-associated pneumonia (VAP), the occurrence of which remains low in children (5, 6).

The optimal definition of EN tolerance/intolerance remains controversial (4), especially the routine measurement of gastric residual volume (GRV) to guide enteral feeding and to reduce the risk of VAP and/or necrotizing enterocolitis in young infants. GRV is defined as the volume of residual feeds and gastric secretions in the stomach, which is measured by aspiration through a gastric tube; it is often considered a surrogate of total gastric content volume, which is composed of residual feeds, gastric secretions, and, sometimes, air (especially in children on respiratory support). In critically ill adults and neonates, the use of GRV to assess EN tolerance has failed to show any benefits but resulted in longer times to reach nutrition goals (7, 8). Laboratory studies further showed that the accuracy of GRV aspiration varied significantly with aspiration technique, feeding tube diameter and material, fluid viscosity, patient position, and position of the tube tip in the stomach (9–11). In critically ill children, no randomized controlled trial has been conducted so far, and practices among pediatric intensive care units vary a lot, as shown in various surveys (12, 13). A retrospective two-center observational comparison study suggested that not measuring GRV in children may not impact outcomes (14).

**Figure 1:**
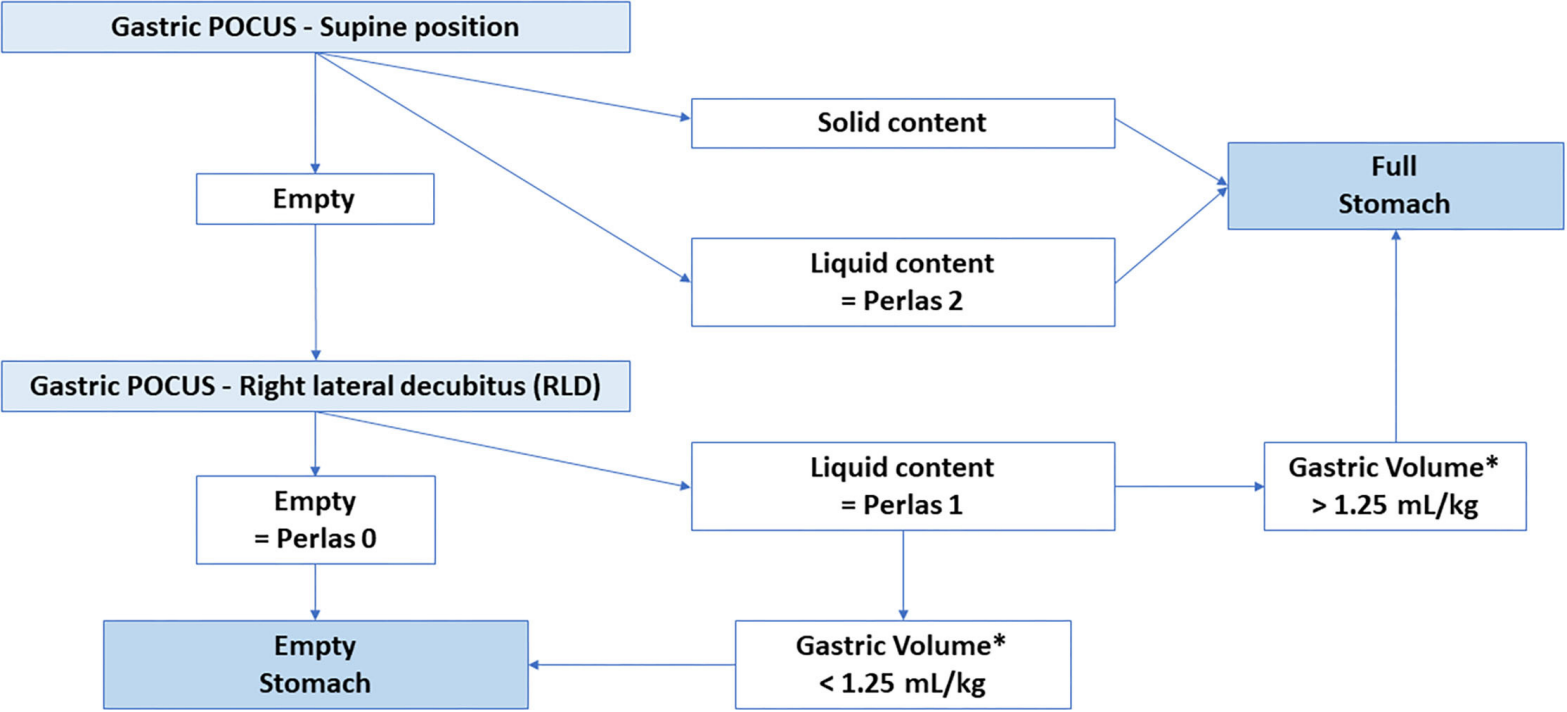
Gastric content according to Perlas and Spencer classifications. POCUS: Point of care Ultrasound *: Spencer et al.: Calculated Gastric Volume (in mL) = −7.8 + (3.5 × RLD CSA) + (0.127 × age) with age in months and RLD CSA in cm^2^.

Gastric point-of-care ultrasound (POCUS) is now part of the algorithm assessing gastric volume and emptiness prior to elective pediatric surgery/sedation, recommended by the European Society of Anesthesiology and Intensive Care (15). It is routinely performed by anesthesiologists based on an ultrasound technique validated by Spencer et al. (16). It adds a quantitative assessment in ml/kg body weight for the gastric contents to the solely qualitative assessment proposed by the adult Perlas classification (17), and allows accurately classifying the stomach as “empty” or “full” (see Figure 1).

Gastric POCUS performed by trained clinicians might be able to be used similarly to assess stomach contents during EN administration in PICU and pre-procedural status of the stomach content. The GastriPed study aimed to assess (using gastric POCUS) the ability of GRV aspiration to empty the stomach and to examine the ability of GRV measurement and gastric POCUS to predict EN tolerance. We hypothesized that <20% of the children would have an empty stomach after GRV aspiration.

## Materials and Methods

We conducted a prospective observational single-center study in a tertiary pediatric intensive care unit (Lyon—France) in 2020–2021. This university hospital PICU admits children aged 0–18 years for both surgical and non-surgical critical illness, but not preterms and post-cardiac surgery patients. Routine GRV measurement was not standard practice in this unit. Children were included if they met the following criteria: from 37-week gestational age to 18 years old, on respiratory support (invasive or non-invasive ventilation), were enterally fed for more than 24 h, and parental consent was obtained. They were excluded if they had had a recent abdominal, esophageal or gastric surgery, if they were fed through a gastrostomy or jejunostomy or if they could not be positioned safely in a right lateral decubitus (RLD) to ensure gastric POCUS measurements. Ethical clearance was obtained (CPP Sud-Ouest et Outre-Mer III: 25/09/2019), and the study protocol was registered on clinicaltrials.gov (NCT04119089) as the GastriPed study. The study is reported according to the STROBE reporting criteria for observational cohort studies (18).

Our main objective was to assess the ability of gastric aspiration (i.e., GRV measurement) to empty the stomach and provide an accurate estimation of gastric volume and emptiness status. Secondary outcomes included the assessment of any relation between GRV orgastric volume (estimated by gastric POCUS) and signs of feeding intolerance, defined as the occurrence of vomiting in the 12 h prior to and after GRV measurement.

**Figure 2:**
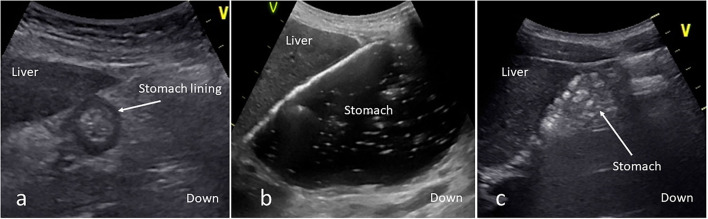
A gastric point-of-care ultrasound [**(a)** empty stomach; **(b)** full stomach with liquid; **(c)** full stomach with solid]. On a sagittal/parasagittal ultrasound scanning plane of the epigastric area, the stomach is visualized under the liver. The “v” mark indicates the probe cursor.

Recruited children underwent gastric POCUS performed by pediatric intensivists who had previously been trained by pediatric anesthesiologists confident with gastric POCUS. A Vivid S6® or a Vivid S70N® (General Electrics, Boston-MA, USA) bedside ultrasound was used with a curvilinear abdominal probe (C2–9 or 4 Hz), or a linear probe (9 Hz). First, children were scanned in a supine position and then in an RLD position. Three measurements of gastric antrum larger (*D*) and shorter (*d*) diameters were performed (to reduce interrater variability-related error) in both positions. This allowed calculating the cross-sectional area (CSA) of the antrum (CSA = *D*/2 × *d*/2 × π) and extrapolating the gastric content volume based on the formula proposed by Spencer et al. (16): Volume (in ml) = −7.8 + (3.5 × RLD CSA) + (0.127 × age) (age in months and RLD CSA in cm^2^). If gastric air interfered with POCUS assessment, gastric content was aspirated through the indwelling gastric tube, air was removed, and liquid gastric content was returned into the stomach. After the first set of gastric POCUS measurements, the gastric content was aspirated by the nurse in charge of the child through the child's gastric feeding tube, using a 50-ml syringe, and GRV was measured and recorded. Correct intra-gastric placement of the tube was confirmed on radiography and/or by measuring the pH of the gastric aspirate. Afterward, gastric antrum measurements were repeated following the same protocol, and corresponding antrum CSA and gastric volume were calculated. In parallel to this quantitative assessment of gastric content, a qualitative assessment was also performed: POCUS operators were asked to describe gastric content as “empty” or “full with liquid,” or “full with both solid and liquid.” (Figure 1). This allowed us to conclude on the emptiness or fullness of the stomach based on Perlas and Spencer classifications (see Figure 2) (16, 17).

Demographic data were recorded from patient electronic health records (i.e., patient body weight and height, age, gender, severity scores PIM2 and PELOD2, length of stay, mechanical ventilation type and duration, main diagnosis, occurrence of necrotizing enterocolitis or VAP). Nutritional data were further collected, i.e., enteral feeding type (breast milk infant feed formula or child feed formula), an enteral nutrition mode of administration (continuous or bolus feeding), gastric tube material and size, and emesis/vomiting occurrence (which were considered as signs of enteral feeding intolerance).

### Data Analysis

Statistical analysis was performed using MedCalc® version 12.1.4.0 for Windows (MedCalc software, Ostend, Belgium). After a D'Agostino χ^2^ test for normality of the continuous data, these were expressed either as means (standard deviation) or medians (interquartile range). Paired measurements (before vs. after gastric aspiration) were analyzed using either the paired sample *t*-test or the Wilcoxon signed rank test as appropriate. Calculated and aspirated gastric contents according to whether vomiting occurred in the 12 h prior to and after GRV measurement were analyzed using either the students' paired *t*-test or the Mann–Whitney *U*-test, as appropriate. Linear regression analysis was performed between the aspirated and the calculated GRV, with calculation of the Pearson correlation coefficient (*r*). Incidence data were expressed as numbers (%) and compared using the χ^2^ test or Fisher's exact test. For each test, *p* < 0.05 was considered as statistically significant. We assumed that 90% of children would have a full stomach at the first ultrasound assessment, and that gastric aspiration would lead to empty stomach in <20% of children. The inclusion of 59 children was sufficient to confirm this assumption, with a significance level of 0.05 and a power of 0.80. We decided to include 65 children to account for the risk of inconclusive ultrasound examinations.

## Results

In total, 65 children were recruited, but one had to be excluded after gastric POCUS measurements because of inability to collect the data (anonymization error). The patients' characteristics are presented in Table 1. The median age (IQR25–75) was 2.95 (0.95–47) months, and weight was 5.3 (3.8–15.5) kg. Nutritional data are presented in Table 2. Signs of EN intolerance occurred in 21/64 (32.8%) of the children.

**Table 1:** Patients' characteristics.

**Characteristics (*N* = 64)**	***N*** **(%) or median (25–75 IQR)**
Age (months)	2.95 (0.95–47.5)
Female gender	26 (41%)
Body weight (kg)	5.3 (3.8–15.5)
Height/length (cm)	58 (52–75)
Length of PICU stay (days)	10.0 (7.0–16.5)
Mechanical ventilation duration (days)	7.5 (5.0–12.0)
Mechanical ventilation (at the time of measurements):	
Non-invasive ventilation	27 (42%)
Invasive ventilation	37 (58%)
PIM2 score	3.8 (1.1–18.9)
PELOD2 score	6 (3-12)
Surgical diagnoses	16 (25%)
Main diagnoses	
Cardiology	2 (3.1%)
Respiratory	31 (48.4%)
Neurology	23 (35.9%)
Gastro-enterology-hepatology	2 (3.1%)
Infectious diseases	3 (4.7%)
Trauma	3 (4.7%)

*PICU, pediatric intensive care unit; PELOD2, pediatric logistic organ dysfunction score 2; PIM2, pediatric index of mortality score*.

**Table 2:** Nutritional data and gastric content assessment.

**Characteristics or measurement (*N* = 64)**	***N*** **(%) or median (25–75 IQR)**
**Enteral feeding type**	
Breast milk (or expressed milk)	18 (28.1%)
Infant feed formula	20 (31.2%)
Child feed formula	26 (40.6%)
Thickened formula	4 (6.2%)
Enteral feeding duration (days)	9.0 (6.0–14.5)
Continuous feeding	62 (96.9%)
**Gastric tube size**	
6 Fr (2 mm)	8 (12.5%)
8 Fr (2.7 mm)	39 (60.9%)
10 Fr (3.3 mm)	11 (17.2%)
12 Fr (4 mm)	5 (7.8%)
14 FR (4.7 mm)	1 (1.6%)
**Gastric tube material**	
Silicon	50 (78.1%)
Polyvinyl chloride (PVC)	14 (21.9%)
Gastric residual volume (ml)	10 (4.5–26.0)
Gastric residual volume (ml/kg)	1.34 (0.79–2.85)
**Feeding intolerance**	
None	43 (67.2%)
Vomiting	21 (32.8%)

**Figure 3:**
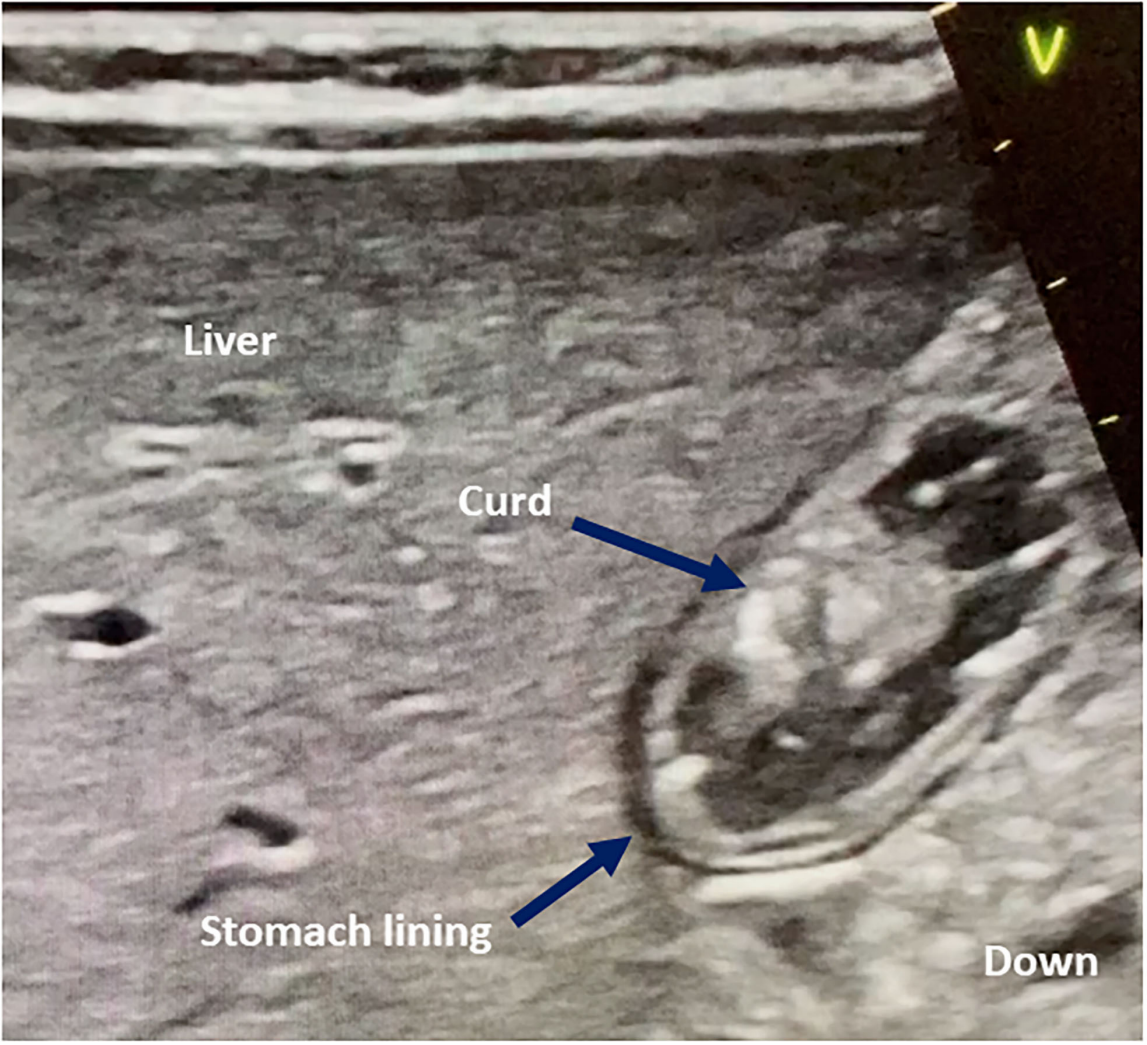
Curd-like gastric content in infants fed with infant formulas or breast milk. On an epigastric sagittal/parasagittal plane, the stomach is visualized under the liver. The “v” mark indicates the probe cursor.

Even if fed with a liquid formula, 7/64 (10.7%) of the children presented with a mixed gastric content (solid and liquid) or solid gastric content (Figure 3), especially in younger infants receiving breast milk. Gastric POCUS measurements were not possible in six children in the supine position because of air interference. All these infants had bronchiolitis and were receiving non-invasive ventilation. Measurements could be performed in the RLD position, although, and Perlas classification was graded 1 or lower.

**Figure 4:**
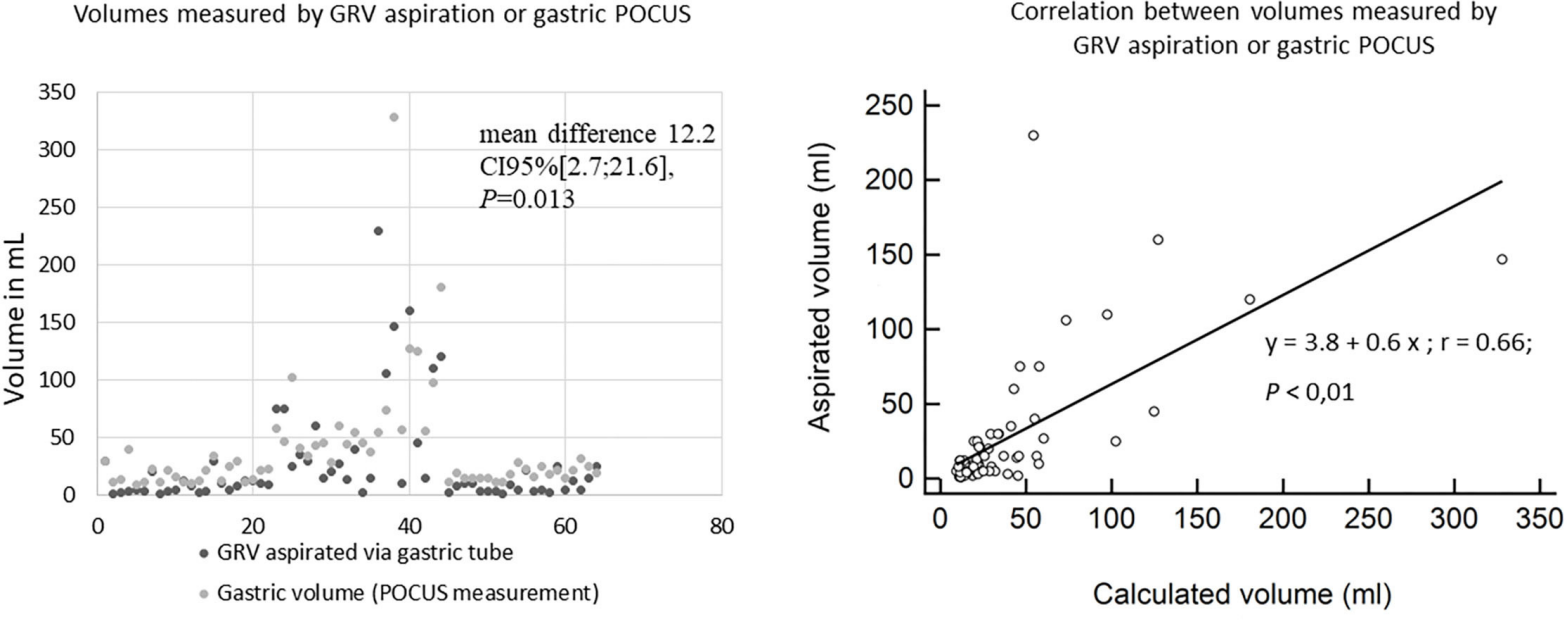
A scattered plot of gastric residual and POCUS volumes for each of the 64 patients (the 64 pairs of measurements are presented along the *X*-axis) and their correlation.

There was a significant difference between pre- and post-GRV aspiration in stomach emptiness, defined as per Perlas (*p* = < 0.001) or Spencer (*p* = < 0.001) classifications and with quantitative gastric POCUS measurements (*p* < 0.001; Table 3). A full stomach was found in 46/64 (71.9%) of the children despite GRV aspiration. Gastric volume POCUS measurement after GRV aspiration showed a large distribution of values, even if expressed in volume per body weight: median, 2.65 ml/kg (IQR 25–75, 1.57–3.57). The calculated gastric volume correlated with the aspirated GRV, which remained lower in almost all cases; the correlation coefficient was *r* = 0.66 (*p* < 0.01; Figure 4).

**Table 3:** Gastric POCUS measurements.

**Measurements [*N* (%) or median (25–75 IQR)]**	**Pre-GRV suctioning**	**Post GRV suctioning**	***P*** **-Value**
Qualitative assessment supine position^a^	*N* = 62	*N* = 62	
Empty	12 (18.8%)	30 (46.9%)	0.002
Full–liquid	46 (71.9)	28 (43.7%)	
Full–solid/liquid	4 (6.2%)	4 (6.2%)	
Qualitative assessment RLD position	*N* = 64	*N* = 64	
Empty	4 (6.2%)	14 (21.9%)	0.039
Full–liquid	54 (84.4%)	45 (70.3%)	
Full–solid/liquid	6 (9.4%)	5 (7.8%)	
Calculated gastric volume (ml)	22.6 (14.6–44.8)	16.5 (11.5–29.8)	<0.0001
Calculated gastric volume per weight (ml/kg)	3.18 (2.40–4.60)	2.65 (1.57–3.57)	<0.0001
Perlas classification	*N* = 64	*N* = 64	
0	4 (6.2%)	15 (23.4%)	<0.0001
1	9 (14.1%)	15 (23.4%)	
2 and solid	51 (79.7%)	34 (53.1%)	
Spencer classification	*N* = 64	*N* = 64	
Empty stomach	5 (7.8%)	18 (28.1%)	0.001
Full stomach	59 (92.2%)	46 (71.9%)	

*GRV, gastric residual volume; RLD, right lateral decubitus*.

a *Supine assessment was not possible in two children because of air interference with gastric POCUS*.

None of the infants were diagnosed with necrotizing enterocolitis or VAP, and 43/64 (67%) of the children presented with no signs of feeding intolerance (vomiting). Neither gastric volume assessed by gastric POCUS prior to GRV nor GRV measurements showed any association with enteral feeding signs of intolerance, when expressed as volume absolute values or as volumes per body weight (Table 4).

**Table 4:** Difference in gastric volume between children presenting with or without enteral feeding intolerance signs (vomiting and or regurgitations).

	**No enteral feeding** **intolerance *N* = 43**	**Enteral feeding** **intolerance *N* = 21**	***P*** **-Value**
Calculated gastric volume (ml)	21.8 (14.5–43.1)	28.0 (15.1–45.5)	0.7
Calculated gastric volume per weight (ml/kg)	3.37 (2.51–4.89)	2.86 (2.19–4.21)	0.19
Gastric residual volume (ml)	9.0 (4.0–22.5)	20.0 (8.0–41.2)	0.14
Gastric residual volume (ml/kg)	1.18 (0.80–2.12)	2.05 (0.78–4.20)	0.14

## Discussion

Our study showed that GRV aspiration reduced the total gastric volume but failed to empty the stomach in a large proportion of children. GRV and gastric content volume calculated by gastric POCUS did not correlate with clinical signs of feeding intolerance.

There is, currently, no consensus definition of enteral nutrition tolerance in critically ill children (19). A systematic review of feeding intolerance in 2019 (4) found it was most frequently defined as the presence of gastrointestinal symptoms (i.e., abdominal distention, diarrhea, and vomiting/emesis) and/or large GRV, or discontinuation of EN due to gastrointestinal symptoms. Feeding intolerance was associated with (rather than correlated) higher severity of illness, healthcare-acquired infections, and mortality. They proposed a definition of feeding intolerance based on the association of insufficient enteral intakes (<2/3 of the daily target energy intake, or EN withheld >48 h) and gastrointestinal symptoms (i.e., large GRV; vomiting, diarrhea, abdominal distention or pain, hematochezia, and melena). However, this current definition has not been agreed by consensus, tested, or validated so far.

GRV has been widely used as a surrogate marker of feeding intolerance and delayed gastric emptying, but its value is increasingly being questioned. Martinez et al. (20), in a study using an acetaminophen absorption test, found that GRV failed to predict gastric emptying and EN administration advancement. This confirms older laboratory studies and clinical study findings (9–11, 21), which suggested that gastric content viscosity, gastric tube size and material, and force of aspiration technique impacted GRV. In our study, GRV aspiration failed to empty the stomach in most cases, especially in breastfed infants in whom curd-like gastric content was identified by gastric POCUS assessment. GRV did not correlate with signs of feeding intolerance, such as vomiting. Most children included in our study, like many children admitted in PICU without gut obstruction, had a soft silicon gastric feeding tube in place, which is different from larger decompression tubes usually made of rigid polyvinyl chloride (PVC). These smaller silicon feeding tubes collapse readily under negative pressure suctioning and thus may significantly reduce the amount of gastric content that can be aspirated, which compromises GRV interpretation.

Gastric POCUS (i.e., performed by non-radiologists at the bedside) has been used in several settings in the last decade, such as pyloric stenosis and gastric foreign body diagnosis by surgeons or emergency department (ED) physicians (22), gastric emptiness assessment prior to sedation by anesthesiologists (23), or gastric tube placement by ED physicians (24). Its learning curve is rapid, and its accuracy was reportedly high. Gastric POCUS was used in our study after a short training period in the study team (three PICU physicians) by the anesthesiologists who use this tool regularly to assess gastric emptiness prior to sedation. Gastric POCUS allowed calculation of the gastric volume using the Spencer formula, which has been validated against the endoscopic assessment of gastric content (16). This confirmed the inaccuracy of GRV in emptying the stomach. No association was found between POCUS gastric volume and enteral feeding tolerance; however, the study was underpowered. This also highlights the fact that gastric content and volume may not be the sole parameter involved in feeding tolerance, and this needs to be confirmed in future studies.

Our study has some limitations that need to be mentioned. The limited study power did not allow drawing conclusions on secondary outcomes (i.e., association between POCUS gastric content and prediction of feeding intolerance). Air interference, especially during NIV support, prevented POCUS operators from obtaining supine images, but RLD assessment remained possible. The inter-operator reproducibility was not assessed prior to the conduct of the study, which may have introduced a measurement error; however, we only used three POCUS-trained pediatric intensivists to perform the gastric POCUS measurements. The learning curve for gastric POCUS has been shown to be rapid and accurate in other settings (25, 26). The Spencer et al. formula that was used to calculate POCUS gastric volume and define gastric content has not been validated in infants, which may have introduced a measurement bias, even if qualitative and quantitative assessments of gastric content were performed in each child prior to and after GRV aspiration (with each child acting as his or her own control). Gastroscopy or MRI would be the gold standard to assess gastric content, but both are invasive and difficult to perform in children. Thus, we chose gastric POCUS as a comparator, which is reliable and non-invasive. Gastric POCUS is now considered relevant in the latest European guidelines on pre-operative fasting when fasting is in doubt or in the case of emergency surgery (15). The GRV measurement technique was not strictly standardized, apart from the use of a 50-ml syringe, as no consensus for this is available (27). Despite these limitations, this is the first clinical study that has compared gastric POCUS with GRV aspiration in patients on PICU.

The practice of GRV measurement to guide feeding is widespread internationally (27), and many PICU health care professionals still believe in it and use GRV measurement, despite the lack of robust evidence to support this practice (12).

To conclude, GRV measurement does not accurately represent stomach contents nor feeding intolerance, as it fails to empty the stomach in most cases. The use of routine GRV measurement to guide enteral feeding may negatively impact clinical outcomes and must be assessed in future PICU trials to confirm the value (or not) of this practice in reducing adverse events (VAP, NEC) on nutritional intakes and on predicting feed tolerance. Gastric POCUS is another surrogate marker of enteral nutrition tolerance and of pre-procedural status of the stomach content, which requires further evaluation before implementing this technique.

## Data Availability Statement

The raw data supporting the conclusions of this article will be made available by the authors, without undue reservation.

## Ethics Statement

The studies involving human participants were reviewed and approved by CPP Sud-Ouest et Outre-Mer III. Written informed consent to participate in this study was provided by the participants' legal guardian/next of kin.

## Author Contributions

FV, LT, and LB designed the study. FV, EC, and CM recruited the patients and performed the gastric POCUS measurements. FV, EC, CM, LT, and LB analyzed the results. LB performed the statistical analyses. FV wrote the draft manuscript, which was reviewed and approved by all authors. LT English edited the manuscript. All authors contributed to the article and approved the submitted version.

## Conflict of Interest

The authors declare that the research was conducted in the absence of any commercial or financial relationships that could be construed as a potential conflict of interest.

## Publisher's Note

All claims expressed in this article are solely those of the authors and do not necessarily represent those of their affiliated organizations, or those of the publisher, the editors and the reviewers. Any product that may be evaluated in this article, or claim that may be made by its manufacturer, is not guaranteed or endorsed by the publisher.
